# Spinal cord atrophy in a primary progressive multiple sclerosis trial: Improved sample size using GBSI

**DOI:** 10.1016/j.nicl.2020.102418

**Published:** 2020-09-09

**Authors:** Marcello Moccia, Nicola Valsecchi, Olga Ciccarelli, Ronald Van Schijndel, Frederik Barkhof, Ferran Prados

**Affiliations:** a Queen Square Multiple Sclerosis Centre, Department of Neuroinflammation, UCL Queen Square Institute of Neurology, Faculty of Brain Sciences, University College London, London, United Kingdom; b Multiple Sclerosis Clinical Care and Research Centre, Department of Neurosciences, Federico II University, Naples, Italy; c National Institute for Health Research, University College London Hospitals Biomedical Research Centre, London, United Kingdom; d Centre for Medical Image Computing, Department of Medical Physics and Bioengineering, University College London, London, United Kingdom; e Department of Radiology and Nuclear Medicine, VU University Medical Centre, Amsterdam, the Netherlands; f Open University of Catalonia, Barcelona, Spain

**Keywords:** Multiple sclerosis, Spinal cord, Atrophy, Clinical trial, GBSI

## Abstract

**Background:**

We aimed to evaluate the implications for clinical trial design of the generalised boundary-shift integral (GBSI) for spinal cord atrophy measurement.

**Methods:**

We included 220 primary-progressive multiple sclerosis patients from a phase 2 clinical trial, with baseline and week-48 3DT1-weighted MRI of the brain and spinal cord (1 × 1 × 1 mm^3^), acquired separately. We obtained segmentation-based cross-sectional spinal cord area (CSA) at C1-2 (from both brain and spinal cord MRI) and C2-5 levels (from spinal cord MRI) using *DeepSeg*, and, then, we computed corresponding GBSI.

**Results:**

Depending on the spinal cord segment, we included 67.4–98.1% patients for CSA measurements, and 66.9–84.2% for GBSI. Spinal cord atrophy measurements obtained with GBSI had lower measurement variability, than corresponding CSA. Looking at the image noise floor, the lowest median standard deviation of the MRI signal within the cerebrospinal fluid surrounding the spinal cord was found on brain MRI at the C1-2 level. Spinal cord atrophy derived from brain MRI was related to the corresponding measures from dedicated spinal cord MRI, more strongly for GBSI than CSA. Spinal cord atrophy measurements using GBSI, but not CSA, were associated with upper and lower limb motor progression.

**Discussion:**

Notwithstanding the reduced measurement variability, the clinical correlates, and the possibility of using brain acquisitions, spinal cord atrophy using GBSI should remain a secondary outcome measure in MS studies, until further advancements increase the quality of acquisition and reliability of processing.

## Highlights

• The GBSI provided clinically meaningful measurements of spinal cord atrophy, with low sample size.
• Deriving spinal cord atrophy from brain MRI using the GBSI is easier than spinal cord MRI.
• Spinal cord atrophy on GBSI could be used as a secondary outcome measure.

## 1 Introduction

Spinal cord atrophy is a common feature of multiple sclerosis (MS), can be detected *in vivo* using MRI, and is one of the main substrates of disease progression (Furby et al., 2010, Rashid et al., 2006, Gass et al., 2015, Rocca et al., 2011). As such, spinal cord atrophy can be used to monitor disease progression, and has been included in clinical trials evaluating medications with putative neuroprotective effects in MS (Brownlee et al., 2017, Kearney et al., 2015, Kearney et al., 2014, Cawley et al., 2018).

In our previous studies (Moccia et al., 2019, Prados et al., 2020), we have adapted the boundary-shift integral (BSI) technique developed for the brain, to be applied to the spinal cord, obtaining the first registration-based method for longitudinal assessment of spinal cord atrophy. This technique, called generalised BSI (GBSI) (Moccia et al., 2019, Prados et al., 2020), significantly reduced measurement error by providing a direct comparison in a joint analysis (i.e., registration of all images in a common space) (Moccia et al., 2019, Altmann et al., 2009), when compared with an indirect comparison of separate segmentations (i.e., numerical subtraction of segmentation values at different timepoints) (Moccia et al., 2019). The latter approach can be compromised by differences in spinal cord coverage, miss-segmentations and/or changes in spinal cord curvature (Prados and Barkhof, 2018, Moccia et al., 2019). Reductions in measurement noise using GBSI could be particularly relevant to clinical trials, that, so far, have failed to show any significant treatment effect on spinal cord atrophy, especially in progressive MS patients (Giovannoni et al., 2020, Ciccarelli et al., 2019), where this outcome measure provides the strongest clinical correlates (Moccia et al., 2019, Ciccarelli et al., 2019).

The previous findings on the use of GBSI need to be confirmed in larger cohorts of progressive MS, including a broader range of clinical and patient-reported outcomes. Additionally, registration-based spinal cord atrophy measurements should be compared at different spinal cord levels and, ideally could be derived also from brain scans. In the present study, we re-analysed a phase 2 clinical trial on primary progressive MS (PPMS) to: 1) compare spinal cord atrophy measurements using segmentation- and registration-based methods, with possible implications for clinical trial design (e.g., measurement variability, image noise floor); 2) compare spinal cord atrophy measurements obtained from routine brain (C1-2) and dedicated spinal cord MRI (C1-2 and C2-5), using segmentation- and registration-based methods; 3) explore possible clinical correlates, also in relation to conventional brain MRI measures; and 4) explore possible treatment effect.

## 2 Methods

### 2.1 Study design

This is a secondary analysis on PPMS patients who participated in the ARPEGGIO phase 2 clinical trial (A Randomized Placebo-controlled trial Evaluating Laquinimod in primary progressive multiple sclerosis, Gauging Gradations In MRI and clinical Outcomes). The ARPEGGIO trial was a randomised, double-blind, parallel-group, placebo-controlled study. Patients were randomised in a 1:1:1 ratio to receive oral Laquinimod 0.6 mg or 1.5 mg, or placebo once daily, from January 2015 to April 2016, at 85 sites in 10 countries. Duration of the core study was 48 weeks (Giovannoni et al., 2020).

### 2.2 Population

Inclusion and exclusion criteria have been reported previously (Giovannoni et al., 2020). Briefly, inclusion criteria were: aged 25–55 years; diagnosis of PPMS (Polman et al., 2011); 3.0–6.5 on the Expanded Disability Status Scale (EDSS) at baseline; documented worsening of clinical disability in the 2 years prior to screening; and a Functional System Score (FSS) ≥ 2 for the pyramidal system or gait impairment due to lower limb dysfunction. Exclusion criteria were: clinical history of any MS exacerbations or relapses; any other neurological disorder (e.g., cervical spinal cord compression, vitamin B12 deficiency); previous use of immunosuppressive/cytotoxic agents, experimental/investigational drugs and/or MS-specific treatments (e.g., Fingolimod, Dimethyl Fumarate, Glatiramer Acetate, Interferon-β, Laquinimod).

### 2.3 MRI acquisition and processing

Sagittal 3D T1-weighted isotropic images (1 × 1 × 1 mm^3^) of the brain and the cervical spinal cord were acquired separately at baseline, week 24 and week 48, within 14 days of the scheduled clinical visit. MRI scans were acquired using scanners from multiple vendors with different field strengths (e.g., 1.5 T and 3 T), and using different acquisition parameters (optimised for each), following site certification; no software/hardware upgrades were allowed between baseline and follow-up scans. MRI scans were quality controlled and collected centrally at the VUmc in Amsterdam. For the purposes of the present study, we included the baseline and week-48 MRI scans.

For the primary analysis of the ARPEGGIO trial, spinal cord area at C1-2 level was determined using the NeuroQLab, a segmentation-based method using a Gaussian mixture modelling (Lukas et al., 2004, Liu et al., 2016, Daams et al., 2014). After definition of the spinal cord subsection to be segmented, watershed segmentation of the spinal cavity and surrounding cerebrospinal fluid was performed, and mean upper cervical spinal cord area (MUCCA) was computed (Daams et al., 2014). Percent change of spinal cord area was calculated using the following formula: MUCCA = 100*(week 48 MUCCA - baseline MUCCA)/baseline MUCCA.

**Fig. 1:**
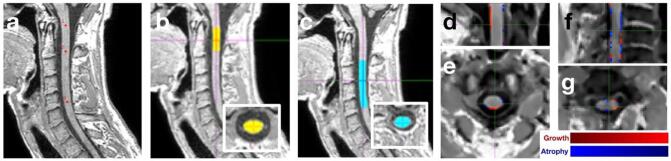
Spinal cord MRI processing using SCT and GBSI. Reference points were manually set at C1, C2 and C5 on sagittal spinal cord scans (a). Then, masks of C1-2 (b) and C2-5 (c) spinal cord levels were obtained from spinal cord images acquired at each time point using the *DeepSeg* tool within the SCT (version 4.0) (sagittal planes are presented, with axial planes in the inset). The probabilistic boundary-shift region-of-interest was then adaptively estimated from baseline and follow-up spinal cord segmentations and the GBSI integral was ultimately computed for C1-2 (d for sagittal plane, e for axial plane) and C2-5 (f for sagittal plane, g for axial plane). CSA: cross-sectional spinal cord area; GBSI: generalised boundary-shift integral; MRI: magnetic resonance imaging; SCT: spinal cord toolbox.

**Fig. 2:**
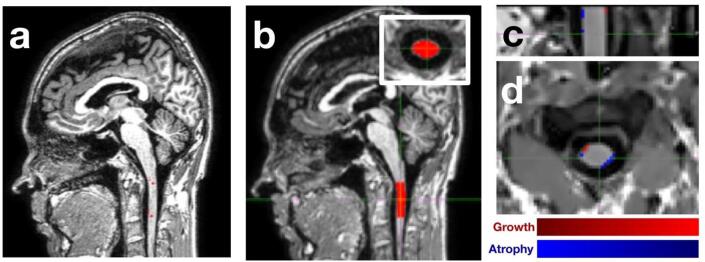
Brain MRI processing using SCT and GBSI. Reference points were manually set at C1 and C2 on sagittal brain scans (a). Then, masks of C1-2 (b) levels were obtained from spinal cord images acquired at each time point using the *DeepSeg* tool within the SCT (version 4.0) (sagittal plane is presented, with axial plane in the inset). The probabilistic boundary-shift region-of-interest was then adaptively estimated from baseline and follow-up spinal cord segmentations and the GBSI integral was ultimately computed for C1-2 (c for sagittal plane, d for axial plane). CSA: cross-sectional spinal cord area; GBSI: generalised boundary-shift integral; MRI: magnetic resonance imaging; SCT: spinal cord toolbox.

In the present study, masks of C1-2 and C2-5 levels were obtained from dedicated spinal cord MRI acquired at each time point, using the DeepSeg tool within the Spinal Cord Toolbox (version 4.0), a fully-automated segmentation method based on convolutional neural networks, which automatically identified different spinal cord levels (Fig. 1) (De Leener et al., 2017, McCoy et al., 2019). Similarly, masks of upper spinal cord (C1-2 level) were obtained from brain MRI, when this part of the anatomy was included in the sagittal acquisition (Fig. 2). Percent change of cross-sectional spinal cord area (CSA) was calculated using the following formula: CSA = 100*(week 48 CSA - baseline CSA)/baseline CSA. For GBSI, we followed the previously described pipeline (Moccia et al., 2019, Prados et al., 2020). Briefly, after straightening the spinal cord at both time points, a 3D symmetric and inverse-consistent affine registration to the half-way space between baseline and follow-up images was performed using 9 degrees-of-freedom (DOF) (translation, rotation and skew, each in three directions); masks were resampled to the same space using linear interpolation to the halfway space. This method does not generate any bias between baseline and follow-up images as the exact same image processing pipeline is applied to both timepoints. The probabilistic boundary-shift region-of-interest was then adaptively estimated from baseline and follow-up spinal cord segmentations. The GBSI was ultimately computed and the percent volume change was measured (Fig. 1; Fig. 2). For subsequent analyses, we excluded scans that showed ± 5% atrophy rate for both CSA and GBSI measurements at a given spinal cord level (e.g., ±5% atrophy rate on both CSA at C1-2 level from brain MRI and GBSI at C1-2 level from brain MRI, on both CSA at C1-2 level from spinal cord MRI and GBSI at C1-2 level from spinal cord MRI, or on both CSA at C2-5 level from spinal cord MRI and GBSI at C2-5 level from spinal cord MRI). The 5% cut-off corresponds to 2 standard deviations in the rate of annual spinal cord loss in healthy controls in our previous study (Prados et al., 2020).

Looking at brain MRI, from the original clinical trial dataset, we extracted number of new T2 lesions, T2 lesion volume change, T1 lesion volume change, and percent brain volume change (PBVC), at baseline and week 48 visits. Full details of acquisitions and processing have been previously reported (Giovannoni et al., 2020).

### 2.4 MRI noise floor

To classify scans based on the noise floor, we used the σ (standard deviation of the MRI signal), calculated with the following formula: $\sigma =\frac{\sigma_{\eta }}{\sqrt{2-\frac{\pi }{2}}}$, where $\sigma_{\eta }$ is the standard deviation of the magnitude–reconstructed cerebrospinal fluid (CSF) signal (Jones and Basser, 2004, Tabelow et al., 2015, Battiston et al., 2018). In particular, the CSF ring surrounding the spinal cord was derived by dilation of the spinal cord mask by 2-pixel layer, and, then, by subsequent subtraction of the mask once; any value of voxels within the extracted ring that were >2 standard deviations above the mean were discarded to avoid the inclusion of values from nerve roots or other spurious signal intensities (Prados et al., 2020, Zhou et al., 2018, Sakaie et al., 2018). The standard deviation of the MRI signal in this CSF ring was calculated to compute the root power of the noise, and, then, used to account for the presence of noise floor. As such, σ provides the scaled signal intensity of the images accounting for the presence of noise floor (rectified noise floor), independently from the magnitude of the CSF signal (Jones and Basser, 2004). This is a commonly applied measure for T1-weighted images, where signal from the CSF is suppressed (expected to be close to 0) (Jones and Basser, 2004), and was automatically computed during GBSI pipeline, for each spinal cord level (Prados et al., 2020). For the purpose of our study, we classified scans based on the median σ for each spinal cord segment.

### 2.5 Clinical variables

Baseline clinical variables were age, sex, disease duration and EDSS.

Using the original clinical trial dataset, we extracted the following clinical variables corresponding to MRI acquisition timing: EDSS, Timed 25-Foot Walk (T25FW), 9-Hole Peg Test (9HPT), Symbol Digit Modalities Test (SDMT), and MS Walking Scale (MSWS). EDSS progression was defined as ≥1 point from baseline EDSS if EDSS at entry was ≤5.5 or increase of ≥0.5 point if EDSS at entry was >5.5. T25FW, 9HPT, SDMT and MSWS progression was defined as ≥20% increase from baseline score.

Assessments were performed by an examining neurologist who remained unaware of the patient's safety status and was instructed not to discuss safety issues with the treating physician, to assure an accurate and objective evaluation (Giovannoni et al., 2020).

### 2.6 Treatment exposure

Patients were randomised in a 1:1:1 ratio to receive oral Laquinimod in a dose of 0.6 mg or 1.5 mg or placebo (once daily). The Laquinimod 1.5 dose arm was discontinued as of January 1, 2016, due to cardiovascular side effects (patients were followed-up, but no further treatment was given). For the purpose of the current analyses, we only included patients with spinal cord MRI at baseline and week 48 visits, whilst early termination visits were excluded; for treatment effect analyses, we only included patients receiving either Laquinimod 0.6 mg or placebo.

### 2.7 Statistical analyses

Baseline demographics and clinical characteristics are presented as mean (and standard deviation), number (and percent), or median (and range), as appropriate.

To evaluate possible implications of different spinal cord atrophy measurements on clinical trial design (aim 1), we computed the sample size required for a hypothetical clinical trial evaluating a neuroprotective medication over a one-year period. Sample size was computed using the formula $n=\frac{{2(Z_{\alpha }+Z_{1-\beta })}^{2}\sigma^{2}}{\Delta^{2}}$, where *n* is the required sample size per treatment arm in 1:1 randomized parallel-groups placebo-controlled trials, *Z_α_* and *Z_1-β_* are kept constant (set at 5% alpha-error and 80% power, respectively), *σ* is the standard deviation (from each spinal cord atrophy measurement), and *Δ* is the estimated treatment effect size (Cawley et al., 2018, Altmann et al., 2009). Effect size was derived from the spinal cord change for each atrophy measurement (percent change of MUCCA, CSA at C1-2 level from brain MRI, GBSI at C1-2 level from brain MRI, CSA at C1-2 level from spinal cord MRI, GBSI at C1-2 level from spinal cord MRI, CSA at C2-5 level from spinal cord MRI, and GBSI at C2-5 level from spinal cord MRI); different treatment effects were simulated (e.g., 30%, 60% and 90%). As additional estimates of measurement variability, we also computed coefficients of variation and median absolute deviations.

To evaluate the possibility of deriving longitudinal spinal cord atrophy measurements from brain MRI (aim 2), we used linear regression models including spinal cord atrophy measurements from brain MRI (percent change of CSA at C1-2 level and GBSI at C1-2 level in turn) and corresponding spinal cord atrophy measurements from spinal cord MRI (percent change of CSA at C1-2 and C2-5 levels, and percent change of GBSI at C1-2 and C2-5 levels, respectively). Results are presented as β-coefficients (Coeff), 95% confidence intervals (95%CI) and p-values from linear regression models.

To evaluate possible clinical correlates of each spinal cord atrophy measurement (aim 3), we used different stepwise linear regression models (one for each MRI variable) with backward selection with *p* = 0.20 as the critical value for entering clinical variables in the model (EDSS progression, T25FWT progression, 9HPT progression, SDMT progression, and MSWS progression). For the purposes of this analysis, we considered both spinal cord MRI variables (percent change of MUCCA, CSA at C1-2 level from brain MRI, GBSI at C1-2 level from brain MRI, CSA at C1-2 level from spinal cord MRI, GBSI at C1-2 level from spinal cord MRI, CSA at C2-5 level from spinal cord MRI, and GBSI at C2-5 level from spinal cord MRI), as well as brain MRI variables (number of new T2 lesions, T2 lesion volume change, T1 lesion volume change and PBVC). To evaluate differences in spinal cord atrophy between Laquinimod 0.6 mg and Placebo (aim 4), we used linear regression models. Results are presented as Coeff, 95%CI and p-values.

As possible confounders of spinal cord atrophy measurements, we included the following covariates to the statistical models: age, sex, height, country and baseline CSA (Oh et al., 2014).

Stata 15.0 was used for data processing and analysis. Results were considered statistically significant when associated with p-values < 0.05.

## 3 Results

### 3.1 Patient disposition

**Fig. 3:**
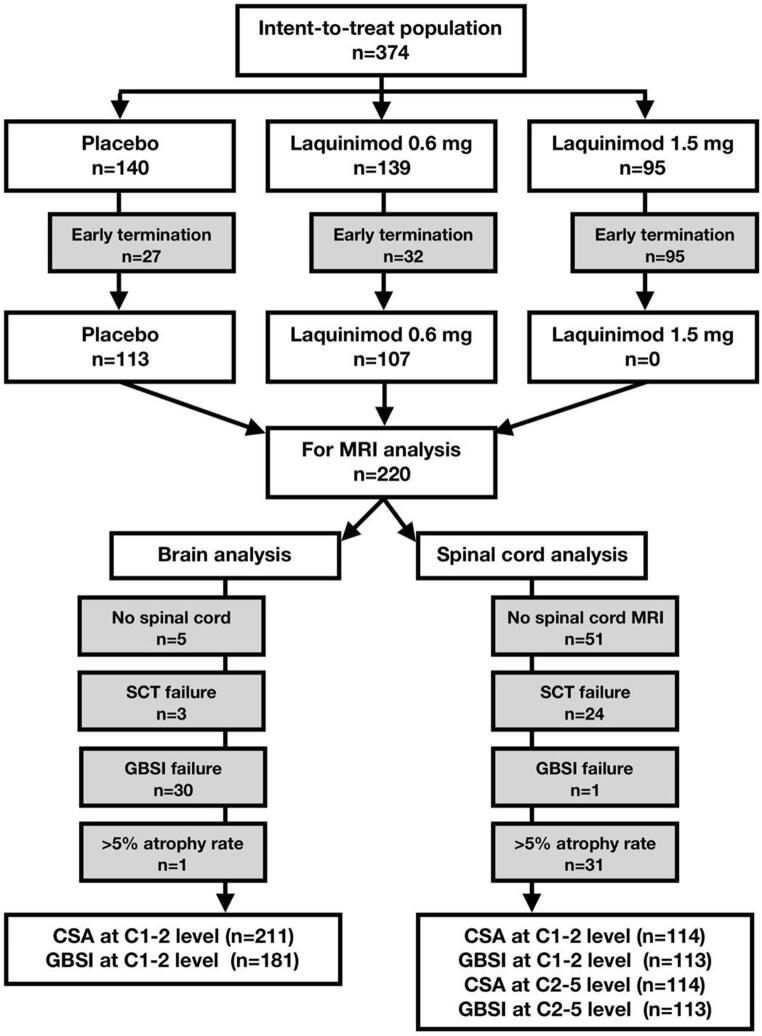
Patient disposition. Patient disposition flow diagram shows number of included and excluded patients. Reasons for exclusion from the original trial population were early termination, lack of upper spinal cord segments included in brain MRI, lack of dedicated spinal cord MRI, SCT failure, GBSI failure, and implausible (>5%) atrophy rates on both CSA and GBSI at each spinal cord level. Total number of patients with each MRI measure is presented. CSA: cross-sectional spinal cord area; GBSI: generalised boundary-shift integral; MRI: magnetic resonance imaging; SCT: spinal cord toolbox.

Patient disposition and reasons for exclusion are reported in Fig. 3. Among patients with baseline and week 48 visits and with MRI acquisitions suitable for analyses, we were able to compute CSA at C1-2 level from brain scans in 98.1% of patients (211/215), in 84.2% of patients for GBSI at C1-2 level from brain scans (181/215), in 67.4% of patients for CSA at C1-2 and at C2-5 level from spinal cord scans (114/169), and in 66.9% of patients for GBSI at C1-2 and at C2-5 level from spinal cord scans (113/169). For comparison, we were had access to data from 220 patients with MUCCA values, number of new T2 lesions, T2 lesion volume change, T1 lesion volume change and PBVC from the original clinical trial.

Demographic and clinical features of included patients are reported in Table 1.

**Table 1:** Demographics and clinical features.

Population (n = 220)	
Age*, years*	46.5 ± 6.8
Sex*, male*	118 (53.6%)
Baseline EDSS	4.5 (3.0–6.5)
Disease duration*, years*	3.4 ± 3.2
Height*, cm*	172.0 ± 9.8
Country*, n*	10

Baseline demographics and clinical features are reported for included patients.

EDSS: expanded disability status scale.

### 3.2 MRI noise floor

**Fig. 4:**
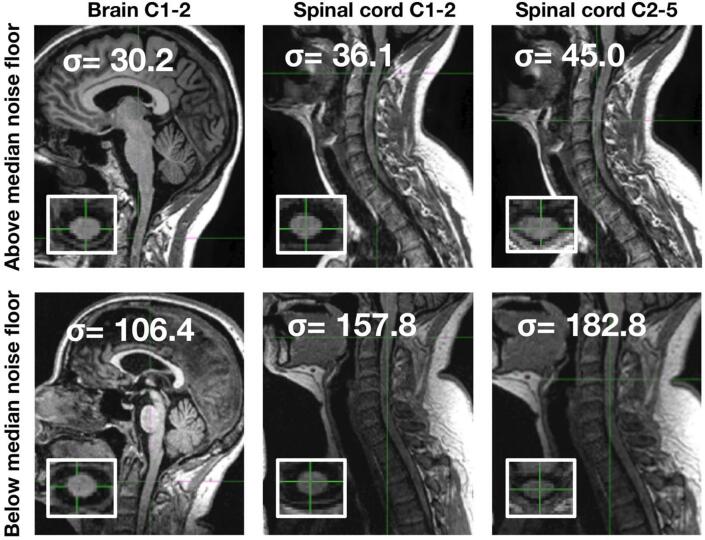
MRI noise floor. Figure shows sagittal plane of brain and spinal cord images above (upper row) and below (lower row) the median noise floor (axial plane in the inset). Different spinal cord levels are reported (C1-2 from brain MRI, C1-2 from spinal cord MRI, and C2-5 from spinal cord MRI). Standard deviation of the MRI signal (σ) within the CSF surrounding the spinal cord is reported for each image. CSA: cross-sectional spinal cord area; GBSI: generalised boundary-shift integral; MRI: magnetic resonance imaging.

Image quality was determined using the median value of σ (standard deviation of the MRI signal within the CSF surrounding the spinal cord) at each spinal cord level. The lowest median standard deviation was found for brain MRI at the C1-2 level (σ = 43.3), followed by spinal cord MRI at the C1-2 level (σ = 57.0), and spinal cord MRI at the C2-5 level (σ = 76.5). Representative images are presented in Fig. 4.

### 3.3 Spinal cord atrophy

**Fig. 5:**
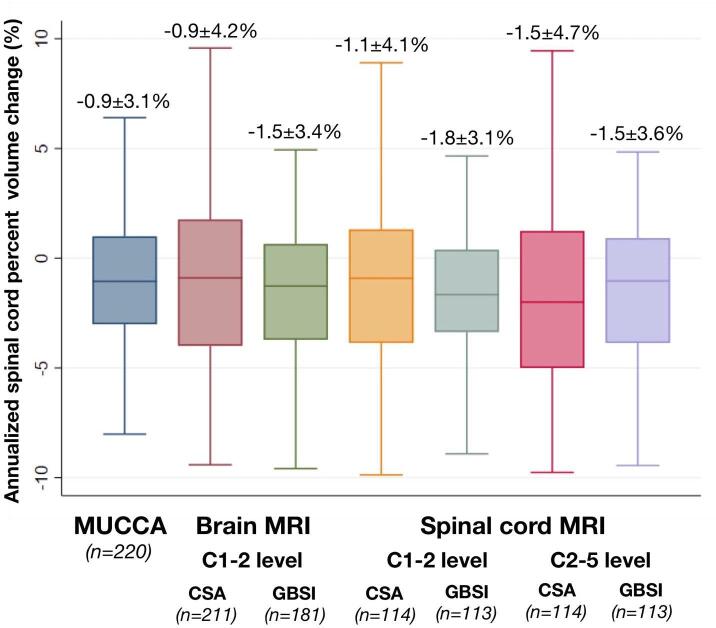
Spinal cord atrophy rates by type of scan and analysis technique. Box-and-Whisker plots show annualised spinal cord percent volume change for the different measurements (percent change from MUCCA, CSA and GBSI analyses at different spinal cord levels from brain and dedicated spinal cord MRI scans). Mean, standard deviation and number of included patients are reported. CSA: cross-sectional spinal cord area; GBSI: generalised boundary-shift integral; MRI: magnetic resonance imaging; MUCCA: mean upper cervical spinal cord area.

Spinal cord atrophy measurements obtained with GBSI (-1.5 ± 3.4% at C1-2 level from brain MRI; −1.8 ± 3.1% at C1-2 level from spinal cord MRI; −1.5 ± 3.6% at C2-5 level from spinal cord MRI) had a slightly larger rate of atrophy and a significantly smaller standard deviation, when compared with corresponding CSA values (-0.9 ± 4.2% at C1-2 level from brain MRI; −1.1 ± 4.1% at C1-2 level from spinal cord MRI; −1.5 ± 4.7% at C2-5 level from spinal cord MRI) and MUCCA values (-0.9 ± 3.1%) (Fig. 5), resulting in smaller sample size estimates, smaller coefficients of variation and smaller median absolute deviations (Table 2). Results were confirmed when considering the subset of patients with MRI scans with a noise floor above the median (Supplementary Material 1a), those with availability of all measurements (Supplementary Material 1b), and those with stable EDSS (Supplementary Material 1a), ruling out selection bias.

**Table 2:** Sample size calculations and measurement variability.

	Total number of patients	Sample size *Treatment effect*			Coefficient of variation	Median absolute deviation
		*30%*	*60%*	*90%*		
MUCCA	220	2073	518	230	−3.56	3.06
CSA at C1-2 level from brain MRI	211	3454	864	384	−4.57	4.13
GBSI at C1-2 level from brain MRI	181	832	208	92	−2.13	3.26
CSA at C1-2 level from spinal cord MRI	114	2528	632	281	−3.42	3.86
GBSI at C1-2 level from spinal cord MRI	113	523	131	58	−1.83	2.83
CSA at C2-5 level from spinal cord MRI	114	1581	395	176	−3.09	4.86
GBSI at C2-5 level from spinal cord MRI	113	1027	257	114	−2.37	3.33

Table shows total number of patients with successfully determined measurements, the sample size estimates in each arm in a randomized clinical trial, coefficients of variation and median absolute deviations using annualised atrophy rates, and median standard deviation for each MRI measure; power was set at 80% and alpha-error at 5%. Different treatment effects were simulated (30%, 60% and 90%). No adjustment for drop-out rates due to clinical reasons or MRI quality were applied. Coefficients of variation and median absolute deviations are also reported.

CSA: cross-sectional spinal cord area; GBSI: generalised boundary-shift integral; MRI: magnetic resonance imaging; MUCCA: mean upper cervical spinal cord area.

### 3.4 Spinal cord atrophy from brain scans

**Fig. 6:**
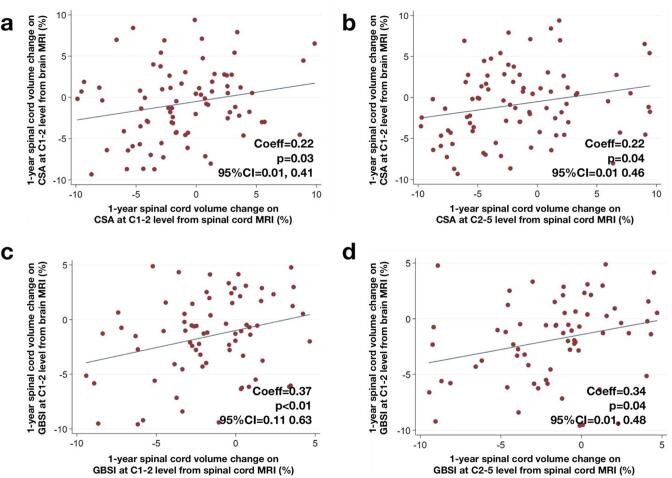
Spinal cord atrophy longitudinal changes from brain and spinal cord MRI. Scatter plots show annualised spinal cord atrophy longitudinal changes derived from brain MRI (CSA at C1-2 level from brain MRI in a and b; GBSI at C1-2 level from brain MRI in c and d) in relation to measurements obtained with the same technique but from dedicated spinal cord MRI (CSA at C1-2 level and CSA at C2-5 level from spinal cord MRI in a and b; GBSI at C1-2 level and GBSI at C2-5 level from spinal cord MRI in c and d). β-Coefficients (Coeff), p-values and 95% confidence intervals (95%CI) are reported from linear regression models. CSA: cross-sectional spinal cord area; GBSI: generalised boundary-shift integral; MRI: magnetic resonance imaging.

Longitudinal spinal cord atrophy measurements obtained from brain MRI scans were related to the corresponding measurements from dedicated spinal cord scans for both CSA (Fig. 6a-b) and GBSI (Fig. 6c-d).

### 3.5 Clinical correlates

Looking at spinal cord atrophy measurements, patients with T25FWT progression presented with more pronounced longitudinal spinal cord atrophy using GBSI at C1-2 level from brain MRI (Coeff = -1.24; 95%CI = -2.30, −0.19; p = 0.02), and GBSI at C2-5 level from spinal cord MRI (Coeff = -1.47; 95%CI = -2.18, −0.75; p < 0.01) (Table 3). Patients with 9HPT progression presented with more pronounced longitudinal spinal cord atrophy using GBSI at C1-2 level from brain MRI (Coeff = -0.92; 95%CI = -1.33, −0.52; p < 0.01) (Table 3). Patients with MSWS progression presented with more pronounced longitudinal spinal cord atrophy using GBSI at C1-2 level from brain MRI (Coeff = -1.86; 95%CI = -2.44, −1.27; p < 0.01), and GBSI at C1-2 level from spinal cord MRI (Coeff = -1.73; 95%CI = -2.38, −1.07; p < 0.01) (Table 3). No significant clinical correlates were detected for longitudinal spinal cord atrophy using MUCCA, CSA at C1-2 level from brain MRI, CSA at C1-2 level from spinal cord MRI, and CSA at C2-5 level from spinal cord MRI (Table 3).

**Table 3:** Clinical correlates of longitudinal spinal cord atrophy.

	EDSS progression		T25FW progression		9HPT progression		MSWS progression		SDMT progression	
	Yes *(23.9%)*	No	Yes *(25.7%)*	No	Yes *(9.2%)*	No	Yes *(15.1%)*	No	Yes *(24.7%)*	No
MUCCA	−1.03 ± 3.59	−0.82 ± 2.94	−1.33 ± 3.02	−0.75 ± 3.10	−1.16 ± 2.35	−0.98 ± 3.05	−0.95 ± 3.19	−0.51 ± 2.74	−1.38 ± 3.09	−0.59 ± 3.07
CSA at C1-2 level from brain MRI	−0.93 ± 4.22	−0.89 ± 4.31	−1.04 ± 4.31	−0.86 ± 4.22	−1.65 ± 5.52	−0.85 ± 4.11	−1.41 ± 4.06	−0.55 ± 4.50	−0.96 ± 4.36	−0.72 ± 4.02
GBSI at C1-2 level from brain MRI	−1.71 ± 3.51	−1.25 ± 3.18	−1.77 ± 3.48*	−1.26 ± 3.36*	−1.68 ± 3.42*	−0.88 ± 3.78*	−1.96 ± 3.46*	−0.72 ± 3.25*	−1.87 ± 3.45	−1.59 ± 3.47
CSA at C1-2 level from spinal cord MRI	−1.39 ± 4.32	−0.53 ± 3.56	−1.55 ± 4.28	−1.11 ± 4.16	−1.27 ± 4.18	−0.71 ± 5.31	−1.24 ± 4.37	−1.20 ± 4.15	−1.23 ± 4.07	−1.03 ± 4.74
GBSI at C1-2 level from spinal cord MRI	−1.89 ± 3.17	−0.96 ± 2.72	−1.84 ± 3.42	−1.63 ± 3.00	−2.15 ± 4.22	−1.61 ± 3.07	−2.21 ± 2.93*	−0.51 ± 3.24*	−1.80 ± 2.86	−1.68 ± 3.21
CSA at C2-5 level from spinal cord MRI	−1.63 ± 4.64	−1.20 ± 5.27	−2.06 ± 4.66	−1.35 ± 4.82	−1.52 ± 4.65	−0.05 ± 7.75	−1.50 ± 5.58	−1.50 ± 4.00	−2.11 ± 5.63	−1.29 ± 4.58
GBSI at C2-5 level from spinal cord MRI	−1.62 ± 3.56	−1.23 ± 3.83	−2.03 ± 4.01*	−1.36 ± 3.50*	−2.69 ± 2.73	−1.45 ± 3.64	−2.15 ± 3.42	−1.22 ± 3.58	−1.84 ± 2.98	−1.14 ± 3.75

Table shows longitudinal spinal cord atrophy in relation to the progression of different clinical outcomes. Percent of patients progressing on each clinical outcome is also shown. * indicates p < 0.05 on different stepwise linear regression models (one for each MRI variable) with backward selection for *p* = 0.20 as the critical value for entering clinical variables in the model.

CSA: cross-sectional spinal cord area; GBSI: generalised boundary-shift integral; MRI: magnetic resonance imaging; MUCCA: mean upper cervical spinal cord area; EDSS: Expanded Disability Status Scale; T25FW: Timed 25-Foot Walk; 9HPT: 9-Hole Peg Test; MSWS: Multiple Sclerosis Walking Scale; SDMT: Symbol Digit Modalities Test.

Looking at brain measurements, patients with EDSS progression presented with increased number of new T2 lesions (Coeff = 2.95; 95%CI = 0.61, 5.29; p = 0.01), higher T2 lesion volume change (Coeff = 1075.53; 95%CI = 479.09, 1671.97; p < 0.01), and more pronounced PBVC (Coeff = -0.40; 95%CI = -0.54, −0.26; p < 0.01). Patients with T25FWT progression presented with higher T1 lesion volume change (Coeff = 142.88; 95%CI = 23.78, 261.97; p = 0.01). Patients with SDMT progression presented with increased number of new T2 lesions (Coeff = 2.66; 95%CI = 0.49, 4.84; p = 0.01), higher T2 lesion volume change (Coeff = 266.56; 95%CI = 21.54, 511.58; p = 0.03), and more pronounced PBVC (Coeff = -0.46; 95%CI = -0.58, −0.33; p < 0.01).

### 3.6 Treatment effect

As previously reported (Giovannoni et al., 2020), the ARPEGGIO clinical trial failed to show any significant treatment effect on percentage brain volume changes (primary outcome measure) (Laquinimod = -0.454% and Placebo = -0.438%; p = 0.90), obtained using Structural Image Evaluation, using Normalization, of Atrophy (SIENA) (Giovannoni et al., 2020). However, Laquinimod and placebo did differ statistically in number of new T2 lesions (0.7 vs 1.6; p < 0.01), T1 lesion volume change (0.0 vs 0.0 mL; p = 0.04), and T2 lesion volume change (0 vs 3 mL; p = 0.01), whilst no differences were detected in rate of EDSS progression (17% vs 23%; p = 0.42), and change in T25FWT score (0.05 vs 0.30 s; p = 0.24). In our current post-hoc analysis, we found that no treatment effect was detected with any of spinal cord atrophy measurements (Table 4).

**Table 4:** Treatment effect on longitudinal spinal cord atrophy.

	Placebo	Laquinimod *0.6 mg*	Coeff	95%CI		P-values
				*Lower*	*Upper*	
MUCCA	−0.81 ± 3.20	−1.38 ± 3.04	−0.25	−1.13	0.62	0.56
CSA at C1-2 level from brain MRI	−1.08 ± 4.10	−1.14 ± 4.12	−0.19	−1.48	1.09	0.76
GBSI at C1-2 level from brain MRI	−1.28 ± 3.40	−2.14 ± 3.51	−0.78	−1.99	0.41	0.19
CSA at C1-2 level from spinal cord MRI	−0.67 ± 4.56	−1.51 ± 4.25	−0.58	−2.55	1.38	0.55
GBSI at C1-2 level from spinal cord MRI	−1.47 ± 2.56	−1.97 ± 3.39	−0.56	−1.97	0.84	0.42
CSA at C2-5 level from spinal cord MRI	−0.80 ± 4.77	−1.80 ± 4.73	−0.42	−2.51	1.66	0.68
GBSI at C2-5 level from spinal cord MRI	−1.31 ± 4.05	−1.51 ± 2.94	−0.38	−2.09	1.32	0.65

Table shows spinal cord atrophy in relation to treatment with Placebo or Laquinimod 0.6 mg. β-Coefficients (Coeff), 95% confidence intervals (95%CI) and p-values are reported from linear regression models adjusted by age, sex, height, country and baseline CSA.

CSA: cross-sectional spinal cord area; GBSI: generalised boundary-shift integral; MRI: magnetic resonance imaging; MUCCA: mean upper cervical spinal cord area.

## 4 Discussion

In the present study, we explored the clinical correlates and implications for clinical trials of segmentation- versus registration-based measurements of spinal cord atrophy in progressive MS, obtained at different cord levels from both routine brain and dedicated spinal cord MRI acquisitions. The registration-based method (GBSI) resulted in clinically meaningful measurements of spinal cord atrophy and relatively low sample sizes, and, thus, could be a candidate secondary outcome measure for clinical trials in MS. Deriving spinal cord atrophy measurements from sagittally acquired volumetric brain MRI scans using the GBSI would allow the use of spinal cord atrophy in multi-centre studies, where acquiring high quality MRI of the spinal cord may be difficult to achieve.

Overall, in line with previous studies comparing CSA and GBSI (Moccia et al., 2019, Prados et al., 2020), we confirm that GBSI measurements provide similar spinal cord atrophy rates (1.5–1.8%/year), when compared with CSA (0.9–1.5%/year) (Moccia et al., 2019, Prados et al., 2020, Casserly et al., 2018), but have smaller related standard deviations, coefficients of variation and median absolute deviations. Due to the reduced measurement variability, GBSI has greater statistical power to detect (treatment) group differences. Improved performance of GBSI is likely the result of its processing pipeline, including straightening, denoising and registration of the spinal cord. In addition, GBSI boundary contours are less affected by (non-random) partial volume effects, which may bias segmentation methods (i.e., inclusion of tissue outside of the area of interest and/or exclusion of tissue within the area of interest), with improved performance (Moccia et al., 2019, Prados et al., 2020, Leung et al., 2010, Tohka, 2014).

We confirmed the strong clinical correlates of spinal cord atrophy (Brownlee et al., 2017, Ciccarelli et al., 2019). In our study, 1-year spinal cord atrophy measurements obtained with GBSI, but not with CSA, at different cord levels (C1-2 and C2-5) and from both brain and spinal cord MRI acquisitions were associated with upper and lower limb motor function, as measured by neurologists (e.g., walking test, and hand test), and by people with MS (e.g., self-reported scale on walking difficulties). Looking at previous longitudinal studies measuring spinal cord atrophy in PPMS, early changes in spinal cord area are associated with clinical changes in the long term (e.g., six to fifteen years) (Tsagkas et al., 2019, Rocca et al., 2017, Aymerich et al., 2018). As such, the lack of association between spinal cord atrophy and EDSS over one year is not surprising, and confirms that spinal cord atrophy should be considered a short term surrogate marker of long-term disease progression (Tsagkas et al., 2019, Rocca et al., 2017, Aymerich et al., 2018). By comparison, brain MRI variables were associated with clinical measures of motor and cognitive disability (e.g., EDSS and SDMT), suggesting they better depict the overall patient status. Of note, we have included only longitudinal brain MRI variables (for both atrophy and lesions), as our interest was in longitudinal spinal cord measures; we therefore did not consider cross-sectional brain or spinal cord volume associations.

An important caveat revealed by the present study is related to difficulties in acquiring good quality spinal cord images. Without specific emphasis on quality of spinal cord scans (secondary outcome in ARPEGGIO), we were unable to extract the relevant outcome measure in 5% to 35% of scans, depending on the spinal cord level, using our automatic pipeline for spinal cord atrophy calculation. The potential failure rate should be accounted for either in the planning phase, by adjusting the sample size accordingly, or by implementing measures to ensure high-quality scan acquisition. Of course, the exclusion of problematic cases from the analysis could have artificially decreased standard deviations and sample sizes for GBSI; however, results did not change when we included the subset of patients with homogeneous quality of scans, with all measurements and with stable EDSS, suggesting that our findings are not biased. In our previous study on combined MAGNIMS and Queen Square spinal cord cohorts, we were able to successfully analyse 85% of scans (Moccia et al., 2019). By contrast, looking at the much older INFORMS clinical trial (Prados et al., 2020), we could classify only 20% of the scans as good quality on visual inspection.

In the present study, failures in SCT and GBSI were related to noise, poor contrast and/or artefacts, that are difficult to account for when using automatic methods for spinal cord segmentation and registration. Indeed, both segmentation (DeepSeg) and registration (GBSI) automatically process MRI scans, and we only visually evaluated the images after processing, for qualitative check of this relatively new method and to evaluate reasons for pipeline failure. We have uniformly excluded observations with ± 5% atrophy rate for both CSA and GBSI, which we have previously identified as biologically implausible (i.e., more than two standard deviations away from the rate in healthy controls) (Moccia et al., 2019, Prados et al., 2020); this approach, though aiming to dismiss statistical outliers (Yang and Hutcheon, 2016, Pollet and van der Meij, 2017), has contributed to a high failure rate and may have slightly inflated statistical power. Factors possibly affecting image quality include gradient nonlinearity, which can be responsible for variability of absolute spinal cord measurements between different vendors (Papinutto et al., 2018, Cohen-Adad, 2020), and depending on placement of the subject in the scanner, as shown in a cross-sectional study (Papinutto et al., 2018). This effect can be reduced by using the Jacobian determinant from nonlinear registrations to correct differences in deformation (Papinutto et al., 2018). We therefore included a symmetric and inverse-consistent registration in the GBSI pipeline, to align the two straightened cords in a half-way space, with each image working as a template for the other (Papinutto et al., 2018). Also, if we assume the presence of vendor-dependent gradient nonlinearity distortion, this would especially affect cross-sectional measurements with absolute spinal cord volume computation. However, for the purposes of our study, we used the percent longitudinal volume change between timepoints, which is ideally less affected by similar vendor-dependent gradient nonlinearity distortion at baseline and follow-up acquisitions, than cross-sectional measurements, since in the ARPEGGIO clinical trial sites were required to acquire baseline and follow-up MRI on the same scanner.

Overall, acquiring good quality images was easier for the brain than the spinal cord, as demonstrated by the variability of the MRI signal within the CSF surrounding the spinal cord (standard deviation increased from 43 on C1-2 level from brain MRI, to 57 and 76 on C1-2 and C2-5 levels on spinal cord MRI, respectively). As such, obtaining spinal cord atrophy measurements from brain scans could represent a viable, efficient and clinically meaningful alternative to the more technically challenging spinal cord image acquisition, in particular in multi-centre settings where homogenous quality of spinal cord acquisitions is difficult to achieve. So far, the possibility to derive spinal cord measurements from brain scans has been successfully explored only in cross-sectional studies (Prados and Barkhof, 2018, Liu et al., 2016, Tsagkas et al., 2018). In our study, notwithstanding the large variability of centres acquiring images with different expertise, protocols and field strength, we found statistically significant, but small, correlation coefficients between measurements derived from brain and cord scans, being higher for GBSI than CSA (0.3 vs 0.2), suggesting that longitudinal spinal cord atrophy from brain scans could better be derived using GBSI than using segmentation-based approaches (e.g., CSA). Differences in noise floor between brain and spinal cord MRI could explain the partial concordance of measurements and could be due to the use of different coils, that can affect image quality at C1-2 level. Indeed, more advanced coils, optimised for both brain and spinal cord, can improve image quality, especially at upper cervical cord level, when compared with conventional coils (Cohen-Adad et al., 2011). Unfortunately, the ARPEGGIO database did not include sufficiently detailed information on coil design at different sites to be accounted for in the statistical models.

Sample size estimates with spinal cord GBSI (e.g., 130–250 per treatment arm, for a 60% effect) are two-to-four fold smaller than CSA (e.g., 400–800 per treatment arm, for a 60% effect), and are on the same order of magnitude as for brain atrophy (the pooled rate of 1-year brain atrophy in placebo- and Laquinimod-treated patients from the ARPEGGIO trial (-0.45 ± 1.00%) corresponds to 215 patients per treatment arm for a 60% treatment effect) (Giovannoni et al., 2020, Moccia et al., 2017). In our previous study on spinal cord GBSI (Moccia et al., 2019), we obtained even smaller estimates for both GBSI (50–100), and CSA (200–800), considering a similar treatment effect and also accounting for physiological spinal cord loss in healthy controls. However, that previous study was conducted in much more selected centres, using MRIs acquired within the Magnetic Resonance Imaging in MS (MAGNIMS) network (Moccia et al., 2019). In the present study, we tried to simulate similar conditions by classifying scans based on the median noise floor; however, when including top quality scans, atrophy variability only partly improved, with increased effect size and reduced standard deviation for some measurements. Thus, future clinical trials aiming to use spinal cord atrophy as a primary outcome measure, rather than simply increase the sample size to account for drop-out rates during analyses, should focus on sites that are able to acquire high quality images. A consensus spinal cord acquisition protocol has been recently developed and tested in about 30 different sites across the world, including different vendors (GE, Siemens and Philips) and could serve as a standard for future multicentre spinal cord MRI studies (http://www.spinalcordmri.org – Protocols). Alternatively, longer follow-up could also be considered (Altmann et al., 2009); unfortunately, in the ARPEGGIO trial, no MRI scans were available beyond week 48.

Finally, we confirmed the lack of treatment effect for Laquinimod on spinal cord atrophy, as in the original ARPEGGIO analysis (Giovannoni et al., 2020). This finding, also in light of the lack of treatment effect on brain atrophy and on disability outcomes (Giovannoni et al., 2020), points towards poor (neuroprotective) efficacy of Laquinimod, rather than measurement issues.

Limitations of the present study include the relatively short follow-up duration (48 weeks). On the one hand, this is in line with phase 2 clinical trial requirements in MS where brain atrophy is the primary outcome measure. However, one-year follow-up is relatively short to acquire sufficiently reliable clinical outcomes (e.g., lack of association with EDSS progression), that could have been better studied in the long term. Also, we used *DeepSeg* for spinal cord segmentation, which is an automated method with high reproducibility, but providing smaller volumes than other methods (e.g., NeuroQLab and Jim) (McCoy et al., 2019, Weeda et al., 2019); we previously ruled out the impact of different segmentation methods on longitudinal spinal cord atrophy measurements on GBSI, but cannot exclude results using the segmentation-based method (CSA) were affected (Prados et al., 2020). Also, we used the standard deviation of the MRI signal within the ring of CSF surrounding the spinal cord to classify scans based on their noise floor (Jones and Basser, 2004, Tabelow et al., 2015), which is a common approach for low signal situations, such as for CSF on T1-weighted images (Prados et al., 2020, Jones and Basser, 2004). This was automatically computed by our pipeline, and represents the variance of the background/CSF signal, independent from its actual scale; other measures (e.g., contrast-to-noise ratio) could be considered in the future, but would require additional manual intervention, which is undesirable for an automated pipeline aiming to be applied in large datasets. Finally, spinal cord acquisitions in ARPEGGIO only included T1 sequences, where outlining lesions is difficult; in the future, the possibility of using PSIR and T2 lesions for GBSI calculation will allow spinal cord lesion segmentation and filling, which can make cord atrophy estimates less variable, as has been shown for brain atrophy (Amiri et al., 2018, Battaglini et al., 2012, Popescu et al., 2014).

## 5 Conclusions

Imaging the spinal cord is challenging, but clinically relevant. Spinal cord atrophy reflects early subtle changes in motor function of the upper and lower limbs, with possibly more obvious clinical correlates in the long-term. Improvements in spinal cord acquisition, processing and analysis (e.g., SCT and GBSI), along with the possibility of deriving spinal cord atrophy measurements from brain MRI scans, can enhance the application of this clinically meaningful imaging outcome measure in multi-centre longitudinal observational studies and clinical trials. Rigorous quality control will be required though, as dedicated spinal cord scans (and the resulting measurements) remain more variable than brain scans. Despite sample size not being far off from those for brain atrophy in the ARPEGGIO study, spinal cord atrophy is destined to remain a secondary outcome for the moment, given the issues around controlling image quality in the spinal cord.

## CRediT authorship contribution statement

**Marcello Moccia:** Conceptualization, Data curation, Formal analysis, Software, Validation, Writing - original draft. **Nicola Valsecchi:** Data curation, Formal analysis, Visualization, Writing - original draft. **Olga Ciccarelli:** Funding acquisition, Investigation, Project administration, Resources, Writing - review & editing. **Ronald Van Schijndel:** Data curation, Investigation, Methodology, Software, Writing - review & editing. **Frederik Barkhof:** Conceptualization, Funding acquisition, Resources, Supervision, Writing - review & editing. **Ferran Prados:** Conceptualization, Formal analysis, Methodology, Software, Validation, Writing - original draft.

## Declaration of Competing Interest

The authors declare the following financial interests/personal relationships which may be considered as potential competing interests: MM has received research funding from ECTRIMS-MAGNIMS, the UK MS Society, and Merck, and honoraria from Biogen, Merck, Novartis and Roche; and consultant fees from Veterans Evaluation Services. NV, and RVS have nothing to disclose. OC has received research funding from UK MS Society, National MS Society, Rosetrees trust and NIHR UCLH BRC. OC serves as a consultant for Biogen, Novartis, Roche, Genzyme, Teva and GE healthcare. FB is supported by NIHR biomedical research centre at UCLH, has received research funding from the Dutch MS Society, and honoraria from Bayer-Schering Pharma, Biogen-Idec, TEVA, Merck-Serono, Novartis, Roche, Synthon BV, Jansen Research, Genzyme, and IXICO. FP has received research grants from Guarantors of Brain, and honoraria from Bioclinica.

## References

[b0005] Altmann D.R., Jasperse B., Barkhof F. (2009). Sample sizes for brain atrophy outcomes in trials for secondary progressive multiple sclerosis. Neurology.

[b0010] Amiri H., de Sitter A., Bendfeldt K. (2018). Urgent challenges in quantification and interpretation of brain grey matter atrophy in individual MS patients using MRI. NeuroImage Clin..

[b0015] Aymerich F.X., Auger C., Alonso J. (2018). Cervical cord atrophy and long-term disease progression in patients with primary-progressive multiple sclerosis. AJNR Am. J. Neuroradiol..

[b0020] Battaglini M., Jenkinson M., De Stefano N. (2012). Evaluating and reducing the impact of white matter lesions on brain volume measurements. Hum Brain Mapp..

[b0025] Battiston M., Schneider T., Prados F. (2018). Fast and reproducible in vivo T1 mapping of the human cervical spinal cord. Magn. Reson. Med..

[b0030] Brownlee W.J., Altmann D.R., Alves Da Mota P. (2017). Association of asymptomatic spinal cord lesions and atrophy with disability 5 years after a clinically isolated syndrome. Mult. Scler..

[b0035] Casserly C., Seyman E.E., Alcaide-Leon P. (2018). Spinal cord atrophy in multiple sclerosis: A systematic review and meta-analysis. J. Neuroimaging.

[b0040] Cawley N., Tur C., Prados F. (2018). Spinal cord atrophy as a primary outcome measure in phase II trials of progressive multiple sclerosis. Mult. Scler..

[b0045] Ciccarelli O., Cohen J., Reingold S. (2019). Spinal cord involvement in multiple sclerosis and neuromyelitis optica spectrum disorders. LancetNeurol.

[b0050] Cohen-Adad J. (2020). Spine Generic Project. Consensus spine-generic acquisition and dataset. Github.

[b0055] Cohen-Adad J., Mareyam A., Keil B. (2011). 32-channel RF coil optimized for brain and cervical spinal cord at 3T. Magn. Reson. Med..

[b0060] Daams M., Weiler F., Steenwijk M.M.D. (2014). Mean upper cervical cord area (MUCCA) measurement in long-standing multiple sclerosis: relation to brain findings and clinical disability. Mult. Scler..

[b0065] De Leener B., Lévy S., Dupont S.M. (2017). SCT: Spinal Cord Toolbox, an open-source software for processing spinal cord MRI data. Neuroimage.

[b0070] Furby J., Hayton T., Altmann D. (2010). A longitudinal study of MRI-detected atrophy in secondary progressive multiple sclerosis. J. Neurol..

[b0075] Gass A., Rocca M.A., Agosta F. (2015). MRI monitoring of pathological changes in the spinal cord in patients with multiple sclerosis. Lancet Neurol..

[b0080] Giovannoni G., Knappertz V., Steinerman A.P. (2020). A randomized, placebo-controlled phase 2 trial of laquinimod in primary progressive multiple sclerosis. Neurology.

[b0085] Jones D.K., Basser P.J. (2004). ‘Squashing peanuts and smashing pumpkins’: How noise distorts diffusion-weighted MR data. Magn. Reson. Med..

[b0090] Kearney H., Rocca M., Valsasina P. (2014). Magnetic resonance imaging correlates of physical disability in relapse onset multiple sclerosis of long disease duration. Mult. Scler..

[b0095] Kearney H., Schneider T., Yiannakas M.C. (2015). Spinal cord grey matter abnormalities are associated with secondary progression and Physical disability in multiple sclerosis. J. Neurol. Neurosurg. Psychiatry.

[b0100] Leung K., Clarkson M., Bartlett J. (2010). Robust atrophy rate measurement in Alzheimer’s disease using multi-site serial MRI: tissue-specific intensity normalization and parameter selection. Neuroimage.

[b0105] Liu X.Y., Lukas X.C., Steenwijk X.M.D. (2016). Multicenter validation of mean upper cervical cord area measurements from head 3D T1-weighted MR imaging in patients with multiple sclerosis. AJNR Am. J. Neuroradiol..

[b0110] Lukas C., Hahn H.K., Bellenberg B. (2004). Sensitivity and reproducibility of a new fast 3D segmentation technique for clinical MR-based brain volumetry in multiple sclerosis. Neuroradiology.

[b0115] McCoy D.B., Dupont S.M., Gros C. (2019). Convolutional neural network–based automated segmentation of the spinal cord and contusion injury: Deep learning biomarker correlates of motor impairment in acute spinal cord injury. AJNR Am. J. Neuroradiol..

[b0120] Moccia M., de Stefano N., Barkhof F. (2017). Imaging outcomes measures for progressive multiple sclerosis trials. Mult. Scler..

[b0125] Moccia M., Prados F., Filippi M. (2019). Longitudinal spinal cord atrophy in multiple sclerosis using the generalised boundary shift integral. Ann. Neurol..

[b0130] Moccia M., Ruggieri S., Ianniello A. (2019). Advances in spinal cord imaging in multiple sclerosis. Ther. Adv. Neurol. Disord..

[b0135] Oh J., Seigo M., Saidha S. (2014). Spinal cord normalization in multiple sclerosis. J. Neuroimag..

[b0140] Papinutto N., Bakshi R., Bischof A. (2018). Gradient nonlinearity effects on upper cervical spinal cord area measurement from 3D T1-weighted brain MRI acquisitions. Magn. Reson. Med..

[b0145] Pollet T.V., van der Meij L. (2017). To remove or not to remove: the impact of outlier handling on significance testing in testosterone data. Adapt Hum. Behav. Physiol..

[b0150] Polman C.H., Reingold S.C., Banwell B. (2011). Diagnostic criteria for multiple sclerosis: 2010 Revisions to the McDonald criteria. Ann. Neurol..

[b0155] Popescu V., Ran N.C.G., Barkhof F. (2014). Accurate GM atrophy quantification in MS using lesion-filling with co-registered 2D lesion masks. NeuroImage Clin..

[b0160] Prados F., Barkhof F. (2018). Spinal cord atrophy rates. Ready for prime time in multiple sclerosis clinical trials?. Neurology.

[b0165] Prados F., Moccia M., Johnson A. (2020). Generalised boundary shift integral for longitudinal assessment of spinal cord atrophy. Neuroimage.

[b0170] Rashid W., Davies G., Chard D. (2006). Upper cervical cord area in early relapsing-remitting multiple sclerosis: cross-sectional study of factors influencing cord size. J. Magn. Reson. Imaging.

[b0175] Rocca M.A., Horsfield M.A., Sala S. (2011). A multicenter assessment of cervical cord atrophy among MS clinical phenotypes. Neurology.

[b0180] Rocca M.A., Sormani M.P., Rovaris M. (2017). Long-term disability progression in primary progressive multiple sclerosis: A 15-year study. Brain.

[b0185] Sakaie K., Zhou X., Lin J. (2018). Retrospective reduction of systematic differences across scanner changes by accounting for noise floor effects in diffusion tensor imaging. Med. Phys..

[b0190] Tabelow K., Voss H.U., Polzehl J. (2015). Local estimation of the noise level in MRI using structural adaptation. Med. Image Anal..

[b0195] Tohka J. (2014). Partial volume effect modeling for segmentation and tissue classification of brain magnetic resonance images: A review. World J. Radiol..

[b0200] Tsagkas C., Magon S., Gaetano L. (2018). Spinal cord volume loss. A marker of disease progression in multiple sclerosis. Neurology.

[b0205] Tsagkas C., Magon S., Gaetano L. (2019). Preferential spinal cord volume loss in primary progressive multiple sclerosis. Mult. Scler..

[b0210] Weeda M.M., Middelkoop S.M., Steenwijk M.D. (2019). Validation of mean upper cervical cord area (MUCCA) measurement techniques in multiple sclerosis (MS): High reproducibility and robustness to lesions, but large software and scanner effects. NeuroImage Clin..

[b0215] Yang S., Hutcheon J.A. (2016). Identifying outliers and implausible values in growth trajectory data. Ann. Epidemiol..

[b0220] Zhou X., Sakaie K.E., Debbins J.P. (2018). Scan-rescan repeatability and cross-scanner comparability of DTI metrics in healthy subjects in the SPRINT-MS multicenter trial. Magn. Reson. Imaging.

