# Supplementary material for: Spinal cord atrophy in a primary progressive multiple sclerosis trial: Improved sample size using GBSI
Source: Neuroimage Clin. 2020 Sep 9;28:102418. doi: 10.1016/j.nicl.2020.102418 (PMC7509079; doi:10.1016/j.nicl.2020.102418)
Supplement: Supplementary Data 1 [file mmc1.docx]

**Supplementary Material 1. Sample size calculations and measurement variability for subsets of MRI noise floor, analysis technique, and clinical features.**

Tables show rate of longitudinal spinal cord atrophy, number of patients for each MRI measure and sample size estimates using 1-year atrophy rate and standard deviation from each MRI measure for different subsets of patients with scans above median noise floor (**a**), with all measurements (**b**), and with stable EDSS (**c**); power was set at 80% and alpha-error at 5%. Different treatment effects were simulated (30%, 60% and 90%). Coefficients of variation and median absolute deviations are also reported.

CSA: cross-sectional spinal cord area; GBSI: generalized boundary shift integral; MRI: magnetic resonance imaging; MUCCA: mean upper cervical spinal cord area.

| 1. **Scans above median noise floor** | **Percent volume** | **Number of patients** | **Sample size**  *Treatment effect* | | | **Coefficient of variation** | **Median absolute**  **deviation** |  |
| --- | --- | --- | --- | --- | --- | --- | --- | --- |
|  | **change** |  | *30%* | *60%* | *90%* |  |  | |
| **MUCCA** | -0.8±3.1% | 110 | 2616 | 654 | 291 | -3.88 | 3.76 | |
| **CSA at C1-2 level from brain MRI** | -0.6±4.2% | 105 | 8537 | 2134 | 949 | -7.00 | 6.64 | |
| **GBSI at C1-2 level from brain MRI** | -1.7±3.6% | 90 | 781 | 195 | 87 | -2.11 | 1.91 | |
| **CSA at C1-2 level from spinal cord MRI** | -1.4±4.0% | 57 | 1422 | 356 | 158 | -2.85 | 2.66 | |
| **GBSI at C1-2 level from spinal cord MRI** | -2.1±3.0% | 56 | 356 | 89 | 40 | -1.42 | 1.13 | |
| **CSA at C2-5 level from spinal cord MRI** | -1.7±4.2% | 57 | 1063 | 266 | 118 | -2.47 | 2.20 | |
| **GBSI at C2-5 level from spinal cord MRI** | -1.6±3.5% | 56 | 834 | 208 | 93 | -2.18 | 2.01 | |

| 1. **All measurements available** | **Percent volume** | **Number of patients** | **Sample size**  *Treatment effect* | | | **Coefficient of variation** | **Median absolute**  **deviation** |  |
| --- | --- | --- | --- | --- | --- | --- | --- | --- |
|  | **change** |  | *30%* | *60%* | *90%* |  |  | |
| **MUCCA** | -0.1±3.0% | 73 | 156800 | 39200 | 17442 | 30.00 | 18.12 | |
| **CSA at C1-2 level from brain MRI** | -0.6±4.6% | 73 | 10240 | 2560 | 1138 | 7.66 | 5.19 | |
| **GBSI at C1-2 level from brain MRI** | -1.9±3.7% | 73 | 661 | 165 | 73 | 1.94 | 1.78 | |
| **CSA at C1-2 level from spinal cord MRI** | -1.2±4.2% | 73 | 2134 | 534 | 237 | 3.50 | 3.16 | |
| **GBSI at C1-2 level from spinal cord MRI** | -1.8±3.2% | 73 | 551 | 138 | 61 | 1.77 | 1.66 | |
| **CSA at C2-5 level from spinal cord MRI** | -1.7±4.4% | 73 | 1167 | 292 | 130 | 2.58 | 2.13 | |
| **GBSI at C2-5 level from spinal cord MRI** | -2.0±3.5% | 73 | 534 | 133 | 59 | 1.75 | 1.54 | |

| 1. **Stable EDSS** | **Percent volume** | **Number of patients** | **Sample size**  *Treatment effect* | | | **Coefficient of variation** | **Median absolute**  **deviation** |  |
| --- | --- | --- | --- | --- | --- | --- | --- | --- |
|  | **change** |  | *30%* | *60%* | *90%* |  |  | |
| **MUCCA** | -0.8±2.9% | 172 | 2289 | 572 | 254 | 3.62 | 3.54 | |
| **CSA at C1-2 level from brain MRI** | -0.9±4.3% | 165 | 3977 | 994 | 442 | 4.77 | 4.13 | |
| **GBSI at C1-2 level from brain MRI** | -1.2±3.1% | 140 | 1163 | 2134 | 129 | 2.58 | 2.46 | |
| **CSA at C1-2 level from spinal cord MRI** | -0.5±3.5% | 91 | 8537 | 392 | 949 | 7.00 | 6.77 | |
| **GBSI at C1-2 level from spinal cord MRI** | -0.9±2.7% | 88 | 1568 | 818 | 174 | 3.00 | 2.86 | |
| **CSA at C2-5 level from spinal cord MRI** | 1.2±5.2% | 90 | 3272 | 437 | 364 | 4.33 | 3.99 | |
| **GBSI at C2-5 level from spinal cord MRI** | 1.2±3.8% | 87 | 1747 | 254 | 194 | 3.17 | 2.87 | |
